# Evolving Interactions and Emergent Functions in Microbial Consortia

**DOI:** 10.1128/mSystems.00774-21

**Published:** 2021-08-24

**Authors:** Alejandra Rodríguez-Verdugo

**Affiliations:** a Department of Ecology and Evolutionary Biology, University of California, Irvine, California, USA

**Keywords:** evolution, microbial ecology, environmental change, species interactions, microbial communities, ecosystem functions, collective metabolism

## Abstract

Microbial communities are constantly challenged with environmental stressors, such as antimicrobials, pollutants, and global warming. How do they respond to these changes? Answering this question is crucial given that microbial communities perform essential functions for life on Earth. Our research aims to understand and predict communities’ responses to change by addressing the following questions. (i) How do eco-evolutionary feedbacks influence microbial community dynamics? (ii) How do multiple interacting species in a microbial community alter evolutionary processes? (iii) To what extent do microbial communities respond to change by ecological versus evolutionary processes? To answer these questions, we use microbial communities of reduced complexity coupled with experimental evolution, genome sequencing, and mathematical modeling. The overall expectation from this integrative research approach is to generate general concepts that extend beyond specific bacterial species and provide fundamental insights into the consequences of evolution on the functioning of whole microbial communities.

## COMMENTARY

Microbial communities perform pivotal functions, from the cycling of elements through Earth’s ecosystems to the digestion of complex foods that shape human health and disease. Many of these functions emerge from the interaction of multiple species working as a collective (1). For example, the degradation of complex polymers in nature often requires a division of labor among species for their breakdown. A key question is how these emergent functions could persist, in the long-term, in face of environmental stressors, such as antimicrobials, pollutants, and global warming. To answer this question, we first need to understand how species and their interactions evolve in response to change.

Achieving such understanding is extraordinarily difficult given that microbial communities are incredibly diverse in terms of numbers of different microbes, genes, and interactions. Progress on this problem requires both top-down studies that deal with the complexity of natural communities and bottom-up research that uses simpler experimental setups (2). My research group works at the intersection between top-down and bottom-up research and strives to understand how microbial communities respond to environmental change and the consequences of these changes on collective functions. By studying simplified communities under both laboratory and field conditions and by addressing fundamental questions, we aim to make conceptual advances in the emerging field of microbial evolutionary ecology.

## HOW DO ECO-EVOLUTIONARY FEEDBACKS INFLUENCE MICROBIAL COMMUNITY DYNAMICS?

Rapid evolution can impact short-term ecological dynamics, and these altered ecological dynamics can feedback to affect subsequent evolutionary change (3). Eco-evolutionary feedbacks have been well documented for pairs of microbes with reciprocal negative interactions, such as predator-prey and host-parasite interactions (4–6). Less is known about how eco-evolutionary feedbacks alter other types of ecological interactions, such as commensalism, in which one species unilaterally benefits another species. This knowledge is critical given that commensal interactions, especially those based on metabolic exchanges (the waste of one species being the substrate for another), underlay important functions, such the breakdown of indigestible polymers in animals (7). One important question is how do commensal interactions based on metabolic exchanges evolve through time and drive microbial community dynamics?

**FIG 1:**
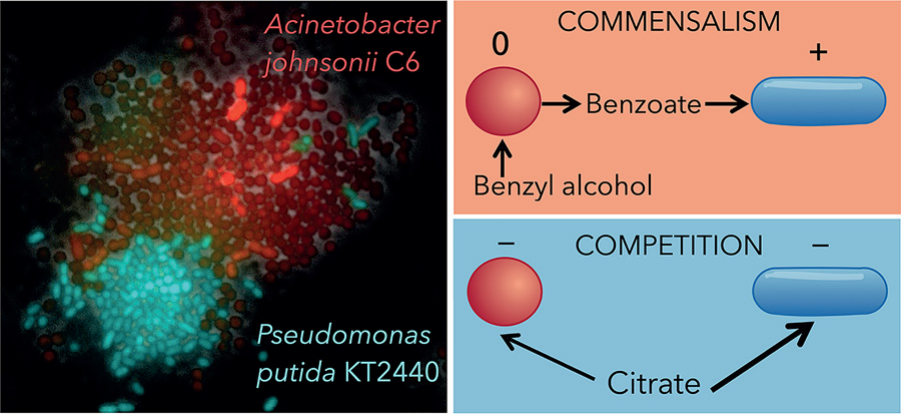
The interaction between *A. johnsonii* and P. putida changes according to the resource provided.

My research has contributed to answering this question by studying the long-term stability of a commensal interaction based on metabolic exchanges. We have been studying the interaction between Acinetobacter johnsonii and Pseudomonas putida, two bacterial species that were originally isolated from a polluted aquifer in Denmark (8). This consortium has been a model system to study commensal interactions for more than 20 years, in part, because species collectively degrade toxic and recalcitrant compounds and therefore can be used for bioremediation (9, 10). Another advantage of working with such a consortium is that species and their interactions are easy to manipulate by controlling the chemical composition of the culture media. Using a simple mathematical model, we showed that interactions are based solely on the use of resources from the external environment (11). We reached this conclusion by building a mathematical model informed by single-species behaviors and by accurately predicting ecological dynamics in a two-species consortium (11). When grown in an environment limited by citrate, species compete for it (i.e., exploitation competition). Instead, when grown in an environment limited by benzyl alcohol, P. putida cross-feeds on the benzoate excreted by *A. johnsonii* (i.e., commensalism) (Fig. 1). Thus, by manipulating the resources, we can manipulate species interactions and address questions related to the long-term stability of different types of species interactions, including interactions that change over time in response to external fluctuations in resources.

To assess the stability of a cross-feeding commensal interaction over evolutionary timescales, we conducted a laboratory evolution experiment over 200 generations using cocultures of *A. johnsonii* and P. putida (12). We found that species rapidly adapted to their culture conditions through *de novo* mutations. Importantly, rapid evolution had different consequences on species coexistence according to the environment. In a constant environment, species stably coexisted, while in a fluctuating environment with daily switches between commensalism and competition, rapid evolution sometimes led to the extinction of *A. johnsonii*. We concluded that eco-evolutionary feedbacks are important drivers of community dynamics in a fluctuating environment but are relatively negligible in a constant environment in which commensalism is the sole type of interaction. Thus, by finding the conditions in which a commensal interaction is stable over evolutionary timescales, we have provided the field of synthetic ecology with a successful example of evolutionary stable consortia (13). The question remains on how cross-feeding commensal interactions could evolve to give rise to new emergent functions (e.g., enhanced biodegradation efficiency). How do species that utilize the waste products of other species ameliorate the collective metabolism of a multispecies system? These questions can be tackled by evolving the Acinetobacter-Pseudomonas consortium with high replication under selective pressures promoting metabolic exchanges. By investigating the fundamental principles governing the evolution of collective metabolism of multispecies systems, we will contribute to explaining the widespread occurrence of cross-feeding interactions (e.g., metabolic dependencies) in natural communities (14, 15).

## DO MULTIPLE INTERACTING SPECIES IN A MICROBIAL COMMUNITY ALTER EVOLUTIONARY PROCESSES?

Although two-species consortia exist in nature and play important roles for ecosystem functioning (16), most microbial communities are composed of more than two species (often containing hundreds or thousands of microbial species). Thus, a major question in microbial evolutionary ecology is whether a focal species responds to selective pressures from one or multiple interacting species (17). In pairwise evolution (coevolution), a population’s evolution is mainly driven by another species: e.g., evolution of antipredator defense. Instead, in diffuse evolution, a population’s evolution is driven by multiple species (18). Determining if evolution is pairwise or diffuse is important for predicting evolutionary trajectories in complex microbial communities. To address this important issue, we are currently assembling simple multispecies communities and plan to assess to what extent the community context (the presence of more than two species) determines the direction and rate of evolution of community members.

## TO WHAT EXTENT DO MICROBIAL COMMUNITIES RESPOND TO ENVIRONMENTAL CHANGE BY ECOLOGICAL VERSUS EVOLUTIONARY PROCESSES?

Microbial communities respond to environmental change through shifts in the identity and frequency of different species (ecological responses) and different genotypes (evolutionary responses). What is the relative contribution of ecological versus evolutionary processes to mediating responses to change? We previously discussed how adaptive evolution mediated by *de novo* mutations can influence ecological dynamics in laboratory settings (12). What remains largely unknown is the contribution of adaptive evolution in communities under natural conditions (19, 20). Filling this knowledge gap is important because evolutionary adaptation may influence key ecosystem functions, such the rates of carbon and nutrient cycling. For example, environmental stressors may lead to the decline or extinction of taxa that are key for carbon cycling. In contrast to these demographic responses, evolution may be able to restore lost functions and even prevent species extinction (e.g., evolutionary rescue). One the other hand, evolutionary adaptation may entail tradeoffs between stress tolerance and other functional traits (21). This example illustrates the importance of identifying mechanisms mediating responses to environmental change to predict consequences for ecosystem functions.

One important ecosystem function is plant litter decomposition, which is performed by microbial communities (i.e., leaf litter microbiomes). I have recently joined a collaboration, The Loma Ridge Climate Change Experiment, aiming to understand how the plant litter microbiome responds to drought in Southern California (22, 23). We are assembling multispecies consortia representative of the leaf litter microbiome from the Loma Ridge grassland site. Our consortia are simple enough that we can easily manipulate them and conduct dozens of evolution experiments in parallel, but are complex enough so that we can capture some of the features of the leaf litter microbiome: e.g., consortia are grown in leaf litter extract in environments with spatial and temporal structures. Importantly, these consortia can be used to conduct manipulative field evolution experiments. In these experiments, consortia are enclosed with litter substrates in microbial cages made of nylon membranes with microscopic pores. This allows the consortia to experience the abiotic conditions from the field while preventing migration from local microbial species (20). These closed systems allow us to easily track changes in species and genotypes frequencies through time (20). Taken together, we expect this approach will bring us one step closer to understanding how communities respond to change in nature and how these responses influence important ecosystems functions.

## FUTURE DIRECTIONS

Using an integrative research approach based on evolution experiments of simple microbial consortia, whole-genome sequencing, and mathematical modeling, we have gained general insights into the mechanisms by which microbial communities respond to environmental change. We are currently working to understand how these responses affect community-level functions important for ecosystem functioning. The next exciting research directions will be (i) to study whether natural selection can act on community-level properties (24) and (ii) to quantify evolutionary rates and mechanisms mediating responses to environmental change under field conditions. By addressing these questions, our research will have lasting impacts on both our understanding of eco-evolutionary dynamics in communities and our current views of the capabilities of organisms in changing environments.
